# The Usefulness of Rare Blood Group Systems in the Risk Determination for Severe COVID-19

**DOI:** 10.3390/pathophysiology28040032

**Published:** 2021-11-03

**Authors:** Theocharis G. Konstantinidis, Valeria Iliadi, Georges Martinis, Maria Panopoulou

**Affiliations:** 1 Blood Transfusion Center, University General Hospital of Alexandroupolis Dragana Campus, 68100 Alexandroupolis, Greece; geomimar@gmail.com; 2 Laboratory of Microbiology, Democritus University of Thrace, University General Hospital of Alexandroupolis Dragana Campus, 68100 Alexandroupolis, Greece; mpanopou@med.duth.gr; 3 Izhevsk State Medical Academy, Kommunarov Street 281, 426034 Izhevsk, Russia; iliadi.valeria@mail.ru

**Keywords:** COVID-19, Forssman antigen, blood type

## Abstract

The newly identified human coronavirus was named severe acute respiratory syndrome coronavirus 2 (SARS-CoV-2), based on a detailed analysis of clinical manifestation. It was reported that blood type O individuals were less likely to become infected by SARS-CoV, while blood type A individuals have an increased risk of severe illness. The Forssman antigen, or Forssman glycolipid synthase (FS), was first described in 1911 by John Frederick Forssman. Blood type A/B glycosyltransferases (AT/BTs) and Forssman glycolipid synthase (FS) are encoded by the evolutionarily related ABO (A/B alleles) and *GBGT1* genes. In this article, based on published studies about the pathogenesis of the COVID-19, we hypothesize the possible relationship between the COVID-19 infection and rare blood type systems, such as the Forssman antigen system.

## 1. Introduction

The newly identified human coronavirus—based on the detailed analysis of clinical manifestation—was named severe acute respiratory syndrome coronavirus 2 (SARS-CoV-2) by the World Health Organization (WHO) and the International Committee on Taxonomy of Viruses (ICTV) [1]. Historically, the study of coronaviruses (CoV) started in the 1970s, with a small researcher community. The first international conference, organized by Volker ter Meulen, met in Wurzburg, Germany in the fall of 1980 [2]. Based on their genomic structure, four main subgroups (alpha, beta, gamma, and delta) were described [3]. CοVs are an enveloped positive-stranded RNA virus. The name was taken for the glycoprotein on their surface arranged in crown-like spikes. All coronaviruses have a spherical structure. The virion consists of transmembrane trimeric spike (S) glycoproteins, a membrane glycoprotein (M), and a transmembrane envelope (E) protein. The RNA is bound to the nucleocapsid protein (N) [4]. The interactions between the SARS-CoV spike protein receptor-binding domain (RBD) and its host cellular receptor regulate both the cross-species and human-to-human transmissions of SARS-CoV [5]. The cellular receptor used by coronaviruses to enter into the host cell differs between different coronaviruses. In 1991, Kathryn Holmes identified the MHV receptor as CEACAM1a (MHVR), a carcinoembryonic antigen family member that is responsible for virus-induced cell fusion [6]. Furthermore, receptors were identified as follows: ACE2 for SARS-CoV, DPP4 for MERS-CoV. It was found that ACE2 is also used by SARS-CoV-2 [7]. 

Human coronaviruses (CoV) cause both upper and lower respiratory tract infections. In the last two decades, two CoVs, named SARS-CoV and MERS-CoV, have been associated with severe human illnesses in 2002 and 2013, respectively [8,9]. The novel coronavirus disease (COVID-19), the severe acute respiratory syndrome coronavirus 2 (SARS-CoV-2), was first reported as cluster of cases in Wuhan, China in December 2019 [10]. On 11 March 2020, the World Health Organization characterized the global health emergency of COVID-19 as a pandemic. As of 27 July 2020, the spread of SARS-CoV-2 had resulted in 16,114,449 confirmed cases globally, with 646,641 registered deaths associated with COVID-19 [11]. SARS-CοV-2 is an enveloped positive-stranded RNA virus like other COVs; however, in contrast to others, it is highly contagious in human-to-human transmission [12].

The clinical manifestation of COVID-19 ranges from asymptomatic infection to acute respiratory distress syndrome with system complications and death [10]. The most common symptoms are fever, cough, expectoration, chest distress, fatigue, and gastroenterological symptoms [10,13]. Moreover, studies showed that COVID 19 triggers coagulopathy, since it is associated with the imbalance of host immune response and endothelial damage [14,15]. 

## 2. The ABO System and COVID-19

It was reported that severe acute respiratory syndrome coronavirus (SARS-CoV) replicates in cells that can express ABO blood type antigens [16]. Moreover, it was indicated previously that blood type O individuals were less likely to become infected by SARS-CoV, while blood type A individuals have an increased risk of severe illness [17]. 

Recently, the Severe COVID-19 GWAS Group identified a gene cluster as a genetic susceptibility locus in patients with COVID-19 and confirmed a potential involvement of the ABO blood type in pathogenesis of disease. They showed a higher risk in blood type A than in other blood types (odds ratio, 1.45; 95% CI, 1.20 to 1.75; *p* = 1.48 × 10^−4^) and a protective effect in blood type O in comparison to other blood groups [18].

The Forssman antigen or Forssman glycolipid synthase (FS), was first described in 1911 by John Frederick Forssman [19]. The gene that codes the FS Ag was discovered in 1996 and was named the *GBGT1* gene [20]. The human *GBGT1* gene is located on chromosome 9 (9q34) and comprises 347 amino acids [21]. This gene has seven exons that span more than 8 kb of DNA, and the coding region is shared by all species that are positive for the FORS Ag. Furthermore, blood type A/B glycosyltransferases (AT/BTs) and Forssman glycolipid synthase (FS) are encoded by the evolutionarily related ABO (A/B alleles) and *GBGT1* genes [21,22]. It was shown that the FS is homologous to α-1,3 GalNAc ABO A transferase (45% sequence identity), and, as a result of this identity, the product of this gene (the FS) cross-reacts with anti-A antibodies [23]. Forssman glycolipid synthetase (FS), is abundantly expressed on the surface of epithelial cells. In addition, van Genderen IL et al. have shown that FS is also localized in subcellular organelles. The Golgi complex, as well as endosomes and lysosomes, were positive for FS. Furthermore, the nuclear envelope was also Forssman positive, but the density was 10-fold less than on the plasma membrane; however, mitochondria and peroxisomes remained free of labels for FS [24]. While the antigenicity of carbohydrates has long been appreciated, when considering the ABO blood groups antigens, the presence of other types of anti-carbohydrate antibodies (ACAs) (such as Forssman antigens (FS Ag)) is poorly understood [25].

## 3. Hypothesis

Based on the published studies about the pathogenesis of COVID-19, we propose a hypothesis concerning the role of the Forssman blood system (Forssman antigen and anti-FORS antibodies) in COVID-19 pathogenesis. 

## 4. Explanation of Proposed Hypothesis

### 4.1. Forssman Synthetase

Forssman synthase (FS) and glycosyltransferase A both result in the addition of the same terminal sugar, α-3-N-acetyl-d-galactosamine, while using different acceptors. The FS adds the terminal sugar to the P antigen, while glycosyltransferase A adds to the H antigen [26]. Previous studies reported that, in humans, the FS gene is presented as an inactive allele [27]. It was also shown that some people express the Forssman antigen due to a reversion in the Forssman synthetase gene. Thus, Forssman-positive individuals lack anti-Forssman antibodies [26]. Furthermore, Yamamoto et al. previously demonstrated that the replacement of the LeuGlyGly tripeptide sequence, from codons 266 to 268 of human AT with GBGT1-encoded FS-specific GlyGlyAla, enabled the enzyme to produce the FORS1 antigen [28]. In place of FS, humans produce the natural anti-Forssman antibody, which activates the complement system when interacting with the FS Ag. These can hemolyze FORS1+ erythrocytes in a complement dependent manner in vitro [29,30].

### 4.2. Forssman Antigen and Diseases

Taniguchi et al. reported that viral infection could lead to enzymatic activity of FS. This hypothesis was assayed in various human lymphoblastic cell lines. The activities of enzymes were found to increase by exposure of the lymphocytes to the Epstein–Barr virus (EBV) [31]. Moreover, the same group had reported that FS activity was higher in patients who suffered from squamous cell carcinomas (in 17 of 18). On the other hand, no increasing enzyme activities were reported in 28 adenocarcinomas [32].

Previous animal models have shown bronchoconstriction, thrombocytopenia, and endothelial cell damage, as well as pulmonary damage, induced by Forssman antibodies [33]. In this context, Nagai et al. reported pulmonary damage and an increase of lactate dehydrogenase (LDH) activity in animals with leukopenia and thrombopenia. Moreover, it has been shown that thromboxane synthase inhibitors reduce the Forssman antisera-induced cell damage [34]. Furthermore, Fan et al. reported that the *GBGT1* gene in the peripheral blood of AMI patients was an upregulated gene in comparison to the control group [35]. In addition, epigenetic mechanisms such as DNA methylation, histone modification, chromatin remodeling, and non-coding RNAs (ncRNA) methylation probably play an important role for FS activation. Cooke describes that *GBGT1* methylation plays a role in pathogenesis of both types of ulcerative colitis [36]. 

## 5. Discussion

Previously, researchers have shown that the ABO antigen’s affiliation, beyond its necessity in daily transfusion medicine, constitutes an independent risk factor of cardiovascular diseases, neoplasm development, and infections [17,37,38,39,40]. Murray et al. report that ABO blood type antigens are expressed in von Willebrand factor (VWF) and that their glycosylation patterns influence circulating VWF levels, and, consequentially, thromboembolic complications [41]. 

The correlation between ABO blood types and the incidence of COVID-19, as well as disease outcomes, has been investigated in several studies [39,42,43]. Pourali et al., in a meta-analysis study, found that blood type A was a partial risk factor for COVID-19 infection, while blood type O was a protective factor [42]. Moreover, Marcos et al. suggest that patients with blood type O have lower susceptibility to infection, in contrast to type B, who have a higher risk of complications [43]. 

Contrary to the ABO system, the clinical significance of the Forssman antigen, as well as anti-FORS antibodies, is still unknown to a large extent. 

It was suggested that differential splicing and/or substitutions of the ABO gene may cause the ABO glycosyltransferase to make the FS structure [21]. Moreover, many blood type A individuals (in particular, A1) do not make anti-FORS antibodies. In addition, the upregulation of the *GBGT1* gene is also implicated in pathogenesis of AMI. Furthermore, based on the knowledge that Forssman synthase uses P antigen as an acceptor, it would be interesting to investigate whether people who do not have P antigen will have a lower susceptibility to infection, similar to people with P phenotype, who are naturally resistant to parvovirus B19 infection [44].

## Data Availability

This data is available from the corresponding author on reasonable request.
